# 

**Published:** 1880-02

**Authors:** 


*CONTENTS.*

| | PAGE |
|---|---|
| COMMON SENSE | 31 |
| DISEASE AND BENEVOLENCE | 34 |
| INSANITY | 35 |
| CIVILIZATION AND HEALTH | 38 |
| CARE OF THE EYES | 43 |
| RULES FOR WINTER | 44 |
| HOW TO CURE A COLD | 45 |
| WHY ? | 46 |
| VENTILATING CHAMBERS | 47 |
| EQUANIMITY OF MIND | 47 |
| A SICK MIND | 48 |
| THERMOMETERS | 49 |
| KEEP YOUR MOUTH SHUT! | 50 |
| CLERICAL EXPOSURES | 51 |
| RESTLESS WANDERERS | 52 |
| BLACKING BOOTS | 53 |
| HEIGHTS | 53 |
| VARIOUS ITEMS | 54 |
| NEWSPAPERS AND PERIODICALS | 56 |
| SLEEPING | 57 |
| SPRING DANGERS | 58 |
| THE NICE YOUNG MAN | 59 |
| WORTH REMEMBERING | 60 |

